# Investigating the effects of digital foot self-management program on enhancing self-efficacy and self-care behavior among community-dwelling older adults with type 2 diabetes: A randomized controlled trial

**DOI:** 10.1177/20552076231220791

**Published:** 2023-12-13

**Authors:** Shu-Ming Chen, Chiung-Jung (Jo) Wu

**Affiliations:** 1 Deputy Dean, 210820 School of Nursing, Fooyin University, Taiwan; 2 School of Health, University of the Sunshine Coast, Australia; 3 Royal Brisbane and Women's Hospital, Australia

**Keywords:** Digital foot self-management program, self-efficacy, self-care behavior, community-dwelling

## Abstract

**Introduction:**

Diabetic foot self-management intervention programs have been proven to positively influence individuals’ behaviors in preventing diabetic foot ulcers. Using digital technologies to deliver programs can facilitate compliance with diabetes self-management programs. However, few studies have focused on the effects of such digital programs on improving the self-efficacy and behaviors of older adults with type 2 diabetes in the community.

**Aim:**

To evaluate the effects of a digital foot self-management program on self-efficacy, self-care behavior, and Hemoglobin A1c levels.

**Design:**

A single-blinded, randomized controlled trial was conducted.

**Methods:**

The intervention program comprised a 4-week digital foot care program with one face-to-face education session, phone calls once weekly, and LINE messages (social media) three times per research nurse and a follow-up of three months. Patients in the control group received routine care.

**Results:**

A total of 100 participants (*n* = 50 in the control and *n* = 50 in the intervention groups) completed the study with a mean age of 67.55 (SD = 11.17). The results showed significant improvements in self-efficacy (*F *= 2187.24, *p *< 0.01) and self-care behavior (*F *= 614.71, *p *< 0.01) in foot care between the groups. The Hemoglobin A1c levels showed a 0.41% reduction over time in the experimental group (*t *= −3.759; *p *< 0.01), whereas the control group showed a 0.06% reduction (*t *= −0.797, *p *> 0.05).

**Conclusion:**

The newly developed digital foot self-management program was effective in community-dwelling older adult patients with type 2 diabetes.

## Introduction

The number of individuals with type 2 diabetes continues to grow and is estimated to reach 700 million by 2045 globally.^1^ The National Health Administration statistics in Taiwan report that 1.76 million people with type 2 diabetes account for 12.3% of the total population in Taiwan. Annually, over 90% of patients with diabetic foot had an infection.^2^ The patients often require recurrining hospital visits, or some patients need to be hospitalized for serious infection over 40 days (Interquartile range: 15–99).^3^ Diabetic foot is the most common, expensive, serious, and preventable complication.^4,5^ The risk of a person with diabetes having foot ulcers over the years is 19%–34%, which can be responsible for 85% of causes of lower limb amputations.^6^

Evidence shows diabetic foot ulcers significantly increase medical costs, reduce quality of life, and increase the risk of premature death.^6^ A higher proportion of patients with diabetic foot ulcers were men, older adults with a low education level, and low income. The prevalence of diabetic foot ulcers was 0.5%–0.8% and showed stable growth year on year in Taiwan.^7^ Approximately 40% of older people with diabetic foot ulcers experience a recurrence within 1 year of ulcer healing, 60% within 3 years of ulcer healing, and 65% within 5 years linked to the complex wound problem of diabetic foot ulcers.^8^ However, at least 70% of amputations caused by diabetic foot ulcers are reported to be preventable by enhancing patient's confidentice in their own self-care behaviors.^9^ Foot care behavior involves feet inspection (such as assessing skin color, temperature, presence of calls), washing and drying, care of toenails, appropriate footwear, and basic wound management.^7,10^

Self-efficacy is a critical construct in Bandura's Social Cognitive Theory.^11^ Self-management programs guided by the self-efficacy model have shown positive improvements in better managing the health outcomes of patients with chronic diseases, such as cardiac and diabetes.^12^ Studies have also shown that providing foot care education based on self-efficacy design to people with diabetes improves foot care behavior.^13,14^ Moreover, diabetes self-management via emerging innovative applications motivates individuals to engage in healthy lifestyle activities to reduce complications. Mobile devices and applications have provided benefits to health professionals by supporting clinicians’ clinical decision-making and improving the health outcomes of people with diabetes.^15^

Multiple digital applications are available for older people with diabetes to more sufficiently self-manage their condition.^16,17^ Digital defines in this study including using mobile devices for accessing calls, videos via application, e-books, which have shown the effect of increasing patients’ willingness to improve their behaviors in managing their everyday conditions.^18^ Patients felt more comfortable and motivated to engage in foot care activities as well as interacting with the research nurse through the application. Despite the many applications available to healthcare providers and patients, evidence evaluating digital foot care interventions specifically designed for older people with diabetic feet is limited.^19^ In addition, lack of theory-based teaching materials targeting in-home patient self-care, we addressed the gap by assisting patients to improve their knowledge and skills. Digital self-care videos and games highlighting the different levels of prevention care were designed so that in-home patients can experience and understand while living with diabetes.

## Aim

This study aimed to develop and evaluate the effectiveness of a digital foot self-management educational program for older adults with type 2 diabetes on self-efficacy, behavior, and hemoglobin A1c (HbA1C) outcomes.

## Methods

### Study design

A two-armed, single-anonymized, randomized controlled trial was conducted to evaluate the effect of a digital foot self-management program. A total of 100 participants were randomly allocated to either the experimental group (*n* = 50) or the control group (*n* = 50). A computerized random number generated by an independent statistician was used to allocate each potential participant to the control or experimental group in a one-to-one ratio. Participants in the experimental group received a digital foot self-management program and routine care. The control group participants received routine care in the diabetes clinic, including routine checkups every three months, and foot care education. Technical support was provided by a research nurse, who liaised with the application developer if necessary. The outcomes were self-efficacy, self-care behaviors during foot care, and HbA1C levels.

### Participants and recruitment

**Figure 1.**
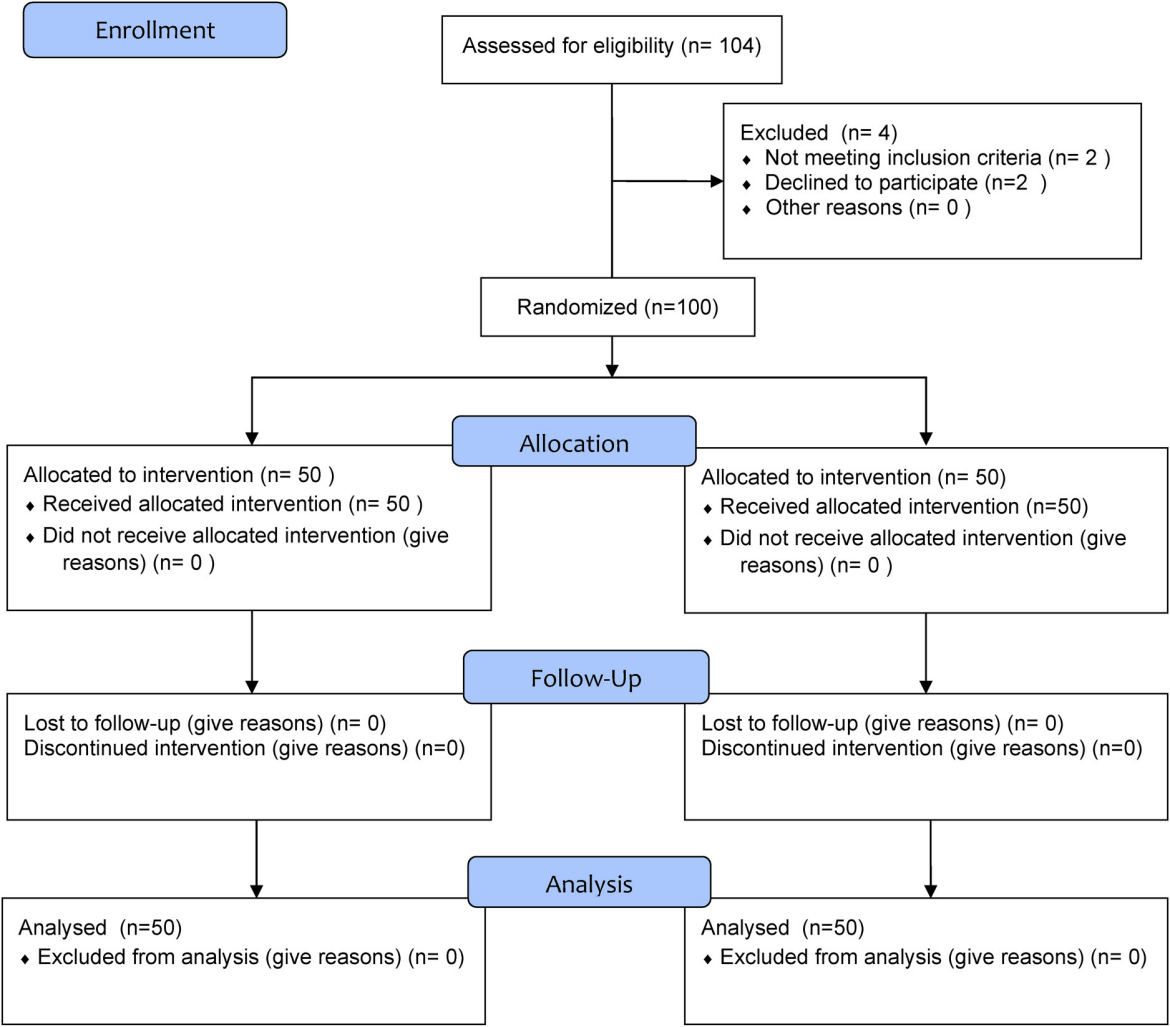
CONSORT flow diagram of the trial.

After obtaining ethical approval and agreement from the community authorities to participate in the study, eligible participants were recruited from five communities in southern Taiwan, and were intermediated by community coordinator. Participants were approached if they met the following inclusion criteria: Diagnosis of type 2 diabetes, age over 65 years, ability to use a mobile device (e.g., smartphone, tablets), and ability to read and comprehend Chinese. Participants were excluded if they could not perform the recommended physical activities, had cognitive impairment, or could not read Chinese (Figure 1).

### Sample size calculation

The sample size calculation was based on the primary outcome of self-efficacy for over 80% of power using G-power 3.1.9.2 to set *F* verification by repeated measured analysis of variance, α=0.05, and the moderate effect size was 0.25, power was 0.80, and 20% attrition rate. Data were collected by a research assistant, and an intervention program was delivered by a research nurse.

### Intervention

A digital foot self-management program guided by Bandura's self-efficacy model^20^ for older people with type diabetes was developed, comprising 4-week sessions and a follow-up of 3 months. The self-efficacy information sources of mastery, vicarious experience, verbal persuasion, and physiological feedback were applied to the program.^11,12^ See Table 1. For example, a peer-supporting video, a quiz game, and a feedback system were used to encourage participants to resolve self-identified programs. The contents were adapted from the diabetes foot care guidelines of The Diabetes Association of Taiwan,^21^ including checking foot conditions and cleaning, assessing for corns and calluses, foot exercise, diet-related foot care, foot care logs, and culture-involved foot care at home. If corns and calluses are identified, patients are asked to see their own doctor for appropriate management. Week 1 of the intervention consisted of a one-hour face-to-face session, introducing key topics and navigating the application functionalities, including an opportunity to discuss and practice activities with the research nurse. During weeks 2–4, participants received weekly follow-up phone calls and three messages through the application feedback system. A research nurse provided an application feedback system for up to 3 months. For the second and third months, participants were encouraged to use the digital platform to monitor their compliance with the program from home. Participants were informed that technical phone support was available during the trial. Benchmarks from digital information literacy on “knowing, accessing, managing information, sharing information”^22^^(p,7)^ were used to assess the participant's digital literacy levels via feedback from engaging in the learning activities.

**Table 1.** Overview of the digital foot self-management program*.

Contents/activities	Self-efficacy model – Information source
Introduction of the intervention by an application	Verbal persuasion
Patient to share the experience via video clips (e.g., checking foot conditions, cleaning, drying, foot exercise etc)	Vicarious experience; self-appraisal
Interaction with research nurse for identifying problem, self-monitoring, and practising foot care activities via interactive foot care games	Mastery; verbal persuasion
Encourage participants to discuss their own experiences	Vicarious experience
Opportunity to have participants questions answered	Vicarious experience; verbal persuasion’ mastery; self-appraisal

* Adapted from Wu & Chang (2014).^11,12,19^

### Outcome measures

Self-efficacy was assessed using the Chinese version of the Foot Care Self-Efficacy Scale.^23^ This scale contains 16 items measuring an individual's foot care management efficacy, and was scored using a five-point Likert scale: “completely disagree,” “slightly disagree,” “no opinion,” “slightly agree,” and “completely agree,” with a minimum of 16 and a maximum score of 80. The Cronbach's alpha was 0.82 in this study. The scale was used at baseline (T1) and at four weeks (T2) for the control and experimental groups.

Self-care behaviors were assessed using the Diabetic Foot Self-Care Behavior Scale.^20^ The scale consists of seven questions divided into two parts consisting of the number of days, and the frequency of foot care activities. On a 5-point Likert scale (never, days a week = 1 point; rarely, 1–2 days a week = 2 points; sometimes, 3–4 days a week = 3 points; often, 5–6 days a week = 4 points; 7 days a week = 5 points). A scale of 7–35 indicates good foot self-care behavior. The Cronbach's alpha was 0.92 in this study. The scale was used at T1 and T2 for the control and experimental groups.

Glycemic control was determined based on HbA1C at baseline and 3 months. Data were obtained from the participants’ medical records.

### Data collection

Data collection commenced after informed consent in paper format, was obtained. Data were collected online through web-connected portals so that trained community nurses could provide individualized foot care and consultation after receiving ethics approval. Demographic data were collected at baseline. Other outcome measures were collected at baseline and 3 months, such as questionnaires and HbA1C in both groups.

### Data analysis

This study was analyzed and calculated using SPSS Windows software version 22.0. Baseline demographic profiles were examined for similarities between the experimental and control groups. Between- and within-group comparisons were performed to examine self-efficacy and self-care behaviors. The Johnson–Neyman technique was conducted to the HbA1C if time and group interaction effects were identified. A statistical of .05 was regarded as a statistical significance.

### Ethical considerations

Ethical approval was obtained from the university hospital. The study was approved by the Ethics Committee (Anonymized for review). Personal information and questionnaire content were anonymous, confidentiality and privacy were maintained for online data collection, and only members of the research team had access to the participants’ app feedback information and physical online data through the principal investigator of this study. These are presented as numbers where necessary. The participants had the right to decide whether to participate or withdraw from the study.

## Results

### Sample characteristics

A total of 100 patients participated in this study. The mean age was 67.55 years (SD = 11.18). The majority were female participants (51%), married (67%), and had completed junior high school (39%). Over 80% were unemployed and had no active foot ulcers (93%). The mean HbA1c was 7.53 (SD = .71). There were no significant differences in the demographics and outcomes at baseline between the experimental and control groups, indicating similarities between the participants in both groups (Table 2).

**Table 2.** Demographic profile in baseline (*n* = 100).

Variable	All *n*(%) Mean ± SD	EG (*n* = 50) *n*(%) Mean ± SD	CG (*n* = 50) *n*(%) Mean ± SD	*t/χ^2^*	*p*
Age(years)	67.55 ± 11.17	67.96 ± 10.80	67.14 ± 11.55	.367	.715
Sex				.360	.715
Male	49(49)	23(46)	26(52)		
Female	51(51)	27(54)	24(48)		
Marital status				1.141	.767
Single	13(13)	6(12)	7(14)		
Married	67(67)	32(64)	35(70)		
Separated/divorced/expired	20(20)	12(24)	8(16)		
Education				7.545	.183
Elementary school (or lower)	23(23)	14(28)	9(18)		
Junior high school	39(39)	18(36)	21(42)		
Senior high school	15(15)	9(18)	6(12)		
University/college (or higher)	23(23)	9(18)	14(28)		
Occupation status				.291	.865
Unemployed	84(84)	42(84)	42(84)		
Employed	16(16)	8(16)	8(16)		
Foot ulcer				.154	.695
Yes	7(7)	4(8)	3(6)		
No	93(93)	46(92)	47(94)		
HbA1C level (%)	75.25 ± 0.74	7.49 ± 0.80	7.56 ± 0.68	−.416	.679
Foot care self-efficacy	24.99 ± 2.593	24.96 ± 2.276	25.02 ± 2.910	−.115	.909
Diabetic foot care behavior	7.94 ± 1.114	8.08 ± 1.140	7.80 ± 1.088	1.256	.212

CG, control group; EG, experimental group; HbA1C: hemoglobin A1c.

### Self-efficacy in diabetic foot care

One-way analysis of variance (ANOVA) showed significant improvements in self -efficacy between intervention and control groups (*F *= 2187.247, *p *< 0.00) indicating that participants who have received digital foot self-management program, have a substantial difference in self-efficacy compared to the control group. Within groups, there was a significant difference between baseline and 4-week intervention in the experimental group (24.96 ± 2.276 vs. 76.56 ± 1.656; *t *= 137.706, *p *< 0.001), but it was significant in the control group (25.02 ± 2.910 vs. 56.0 ± 2.597; *t *= 65.352, *p *< .001). See Table 3.

**Table 3.** Effects of foot care self-efficacy and behavior from baseline to 4 weeks in the two groups (*n *= 100).

Variation	*SS*	*Df*	*MS*	*F*	*p*	*η_p_^2^*
Foot care self-efficacy						
Baseline (covariance)	18.294	1	18.294	3.974	0.049	0.039
Groups	10,068.690	1	10,068.690	2187.247	0.000*	0.958
Error (between-group)	446.526	97	4.603			
Diabetic foot care behavior						
Baseline (covariance)	1.959	1	1.959	0.453	0.503	0.005
Groups	2658.847	1	2658.847	614.710	0.000*	0.864
Error (between-group)	419.561	97	4.325			

### Diabetic foot care behavior

ANOVA showed significant improvements in diabetes foot care behavior between intervention and control groups (*F *= 2.99, *p *= 0.087) indicating that participants who have received digital foot self-management program, have a substantial difference in self-efficacy compared to the control group. Within groups, there were significant differences between baseline and 4-week intervention in the experimental group (8.08 ± 1.140 vs. 32.36 ± 1.453; *t *= 100.122, *p *< .001) and also significant in the control group (7.80 ± 1.088 vs. 22.00 ± 2.548; *t *= 33.895, *p *< .001). See Table 3.

### Glycemic control

**Figure 2.**
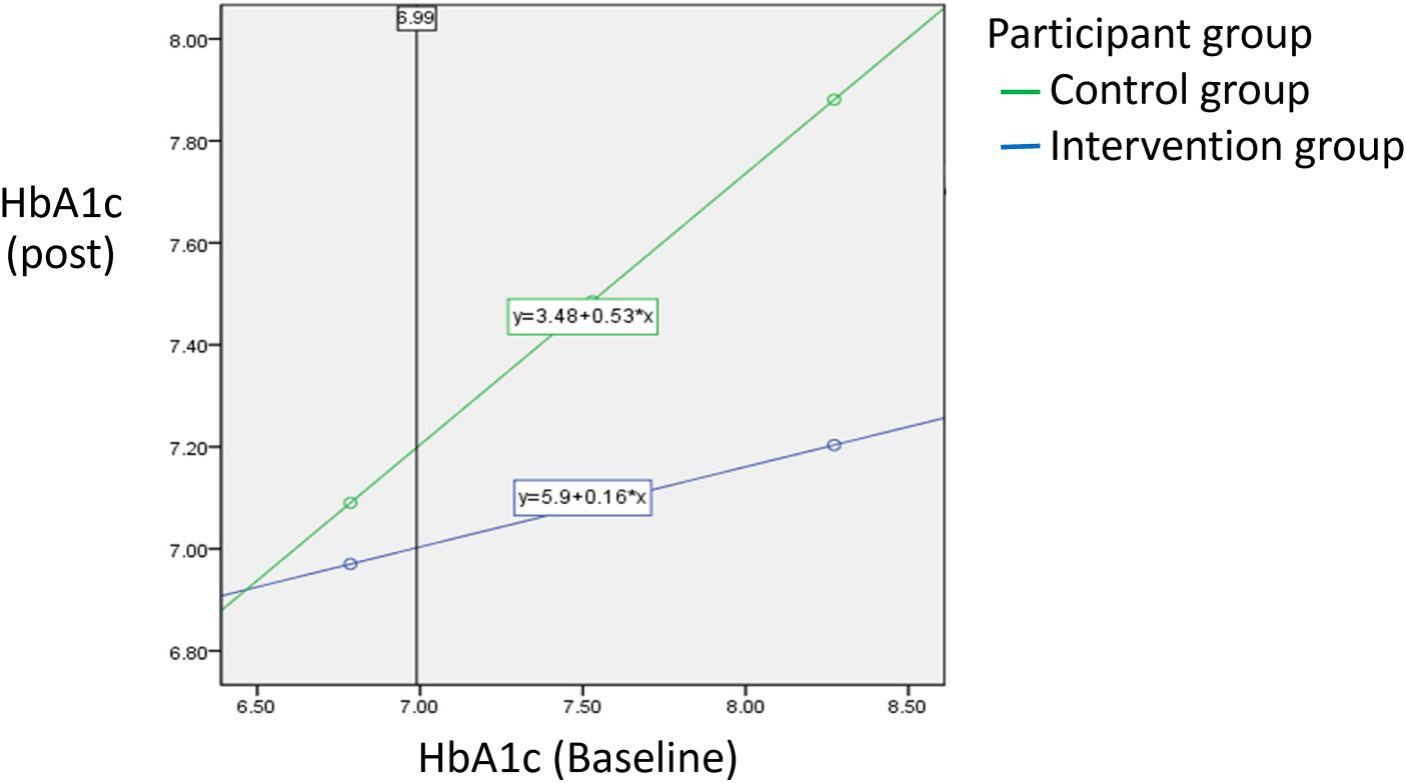
The Johnson–Neyman statistical analysis of HbA1cC, baseline data in each group and the cut points of significant differences between the two groups.

Glycated hemoglobin (HbA1C) levels were measured at baseline and 3 months. The mean HbA1C levels in the experimental group reported a 0.41% reduction (7.49 ± 0.80 to 7.08 ± 0.41) (*t *= −3.759; *p *< 0.001) over time. The control group showed no significant HbA1C level increase of 0.06% from baseline to 3 months, respectively (7.56 ± 0.68 to 7.50 ± 0.54) (*t *= −0.797, *p *= 0.429). In comparison, the independent *t*-test for the experimental and control groups at 3 months showed a significant difference (*t *= −4.35, *p *< 0.001). However, because the interaction between the group and baseline HbA1c was significant (*F *= 11.575; *p *< 0.05), the Johnson–Neyman technique, rather than independent samples one-way ANCOVA was used to explore the intervention effect on HbA1C. The results showed digital foot self-management program significantly improved HbA1C among participants, with baseline values greater than 6.99%. The improvements were more significant in the experimental group than in the control group (Figure 2).

## Discussion

Our newly developed digital foot self-management program positively affected the outcomes of self-efficacy, self-care behavior, and HbA1C levels. There are few digital foot care programs in the studies developed for community-based diabetic foot care in older adults, one of which is Dincer and Bahçecik.^24^ Dincer and Bahçecik developed a mobile application on foot care for patients and a population management system for a hardware device that synchronizes patients’ footcare animation-supported health literacy from their mobile devices. Their mobile device incorporated aspects of care and was used for educational purposes. Our findings support using digital platforms as an intervention delivery model to reduce health professionals’ direct contact time and allow them more time to attend to others’ needs. The findings comprise literature that used the digital platform to promote patients’ awareness, and increase their self-efficacy and behavior to care for their own feet, reducing nursing manpower and medical costs.^18^ We acknowledge there are other applications for the self-management of the diabetic foot in terms of exercises and pressure sensing.^24^ Our theory-based intervention via a digital platform was specifically designed for older people with diabetic foot ulcers in the 40% community.

Patient health literacy and age remained crucial components of optimizing the effect of digital foot care.^22,25^ Motivating engagement and changing foot care behavior in older people with lower health literacy can present challenges for health professionals. Previous studies have shown that patients with improved self-efficacy would feel more confident about managing their diabetic foot care.^26^ Our study used a digital foot self-management program with self-help games designed for a detailed analysis with a Chinese cultural design platform to help participants understand their own situation and care. One of the critical contributions to our intervention program was evidence-based self-care activities, such as checking feet daily, walking barefoot, checking the water temperature before bathing, and self-rubbing for foot woods.^10^ Our results highlight the importance of considering the environment in which people live. With the high humidity weather in the summer in Taiwan, some individuals often walk barefoot at home, which puts them at risk of foot ulcers.

Barriers and facilitators in using digital applications in healthcare have been investigated. Facilitators include the use of digital health programs by health professionals, specific group, or geographic locations could potentially enable the utilizations. However, infrastructure, training, time, environment factors could influence the uptake of digital interventions.^27^ Environmental factors highlight the challenges of managing foot ulcers, which can be affected by wound healing and patient foot care behaviors.^27,28^ Anecdotally, our participants were initially less or not interested in foot care. Nonetheless, after receiving the intervention program, they said, “It is not as difficult as I thought it would be.” “I can do it again.”

### Strengths and limitations

This study's major strength was the program's context, which used the self-efficacy model to guide the digital design of older people with diabetes in the community. However, this study had some limitations. This study was focused on the Taiwanese population's specific environment and culture for diabetic foot care, it may be appropriate to generalize to other population. Furthermore, although significant improvements in glycemic controls have been found in our study, it is unknown as whether how long these older people with diabetic foot care would continue to be embedding in their everyday activities. We also acknowledge that the limitation of not being able to record individual's interaction time with the application and its association with the outcome.

### Future research

The implications for community diabetic foot care include incorporating a digital foot self-management program into existing diabetes education. A longitudinal study is recommended to address these limitations. Future research on a holistic approach that seeks and incorporating feedback from all stakeholders (patients, health professionals, community members, administrators) is also recommended. Future study on the dose-effect of the digital intervention program is suggested.

## Conclusion

Our study was the first randomized controlled trial of a digital foot self-management program for older people with type 2 diabetes living in a community in Taiwan. The results highlighted the benefits of a theoretical model for facilitating people's self-management of their conditions at home.

## References

[bibr1-20552076231220791] Miranda C Da Ros R Marfella R . Update on prevention of diabetic foot ulcer. Arch Med Sci Atheroscler Dis, 2021; 6: e123–e131. 10.5114/amsad.2021.107817 PMC8336435 34381913

[bibr2-20552076231220791] Ministry of Health and Welfare [Internet]. Health statistics. Taiwan; 2019 [cited 22 Nov 5 2022]. https://www.mohw.gov.tw/np-126-2.html

[bibr3-20552076231220791] Tai CH Hsieh TC Lee RP Lo SF . Prevalence and medical resource of patients with diabetic foot ulcer: A nationwide population-based retrospective cohort study for 2001-2015 in Taiwan. Int J Environ Res Public Health 2021; 18: 1891. 10.3390/ijerph18041891 33669255 PMC7920020

[bibr4-20552076231220791] Sathya K Karthi R . A study to assess the effectiveness of Buerger-Allen exercise to prevent risk of diabetic foot by improving lower extremity perfusion among clients with type-2 diabetes mellitus in selected hospitals at villupuram district, Tamilnadu. Int J. Inf Res Rev 2019; 6: 83–88.

[bibr5-20552076231220791] Hinchliffe RJ Forsythe RO Apelqvist J , et al. International Working Group on the Diabetic Foot (IWGDF). Guidelines on diagnosis, prognosis, and management of peripheral artery disease in patients with foot ulcers and diabetes (IWGDF 2019 update). Diabetes Metab Res Rev 2020; 36: e3276. 10.1002/dmrr.3276 31958217

[bibr6-20552076231220791] Edmonds M Manu C Vas P . The current burden of diabetic foot disease. J Clin Orthop Trauma 2021; 17: 88–93. doi: 10.1016/j.jcot.2021.01.017 33680841 PMC7919962

[bibr7-20552076231220791] Lin KD Hsu CC Ou HY , et al. Diabetes-related kidney, eye, and foot disease in Taiwan: An analysis of nationwide data from 2005 to 2014. J Formos Med Assoc 2019; 118: S103–S110. DOI: 10.1016/j.jfma.2019.07.027 31477486

[bibr8-20552076231220791] Allen L Powell-Cope G Mbah A , et al. A retrospective review of adverse events related to diabetic foot ulcers. Ostomy Wound Manag 2017; 63: 30–33. 28657897

[bibr9-20552076231220791] Miranda C Da Ros R Marfella R . Update on prevention of diabetic foot ulcer. Arch Med Sci Atheroscler Dis 2021 6: e123–e131. doi: 10.5114/amsad.2021.107817 PMC8336435 34381913

[bibr10-20552076231220791] Schaper NC van Netten JJ Apelqvist J , et al. IWGDF Editorial board. Practical guidelines on the prevention and management of diabetes-related foot disease (IWGDF 2023 update). Diabetes Metab Res Rev 2023: e3657. doi:10.1002/dmrr.3657 37243927

[bibr11-20552076231220791] Bandura A . Health promotion by social cognitive means. Health Educ Behav 2004; 31: 143–164. 10.1177/1090198104263660 15090118

[bibr12-20552076231220791] Wu C-J Chang AM . Application of a theoretical framework to foster a cardiac-diabetes self-management programme. Int Nurs Rev 2014; 61: 336–343. http://onlinelibrary.wiley.com/doi/10.1111/inr.12104/abstract 24847741 10.1111/inr.12104

[bibr13-20552076231220791] Seyyedrasooli A Parvan K Valizadeh L , et al. Self-efficacy in foot-care and effect of training: a single-blinded randomized controlled clinical trial. Int J Community Based Nurs Midwifery 2015; 3: 141–149. 26005694 PMC4441354

[bibr14-20552076231220791] Toygar İ Hançerlioğlu S Utku T , et al. Effect of an educational intervention based on bandura's theory on foot care self-efficacy in diabetes: a prospective quasi-experimental study. Int J Low Extrem Wounds 2022; 21: 414–419. 10.1177/1534734620948327 32806981

[bibr15-20552076231220791] Fleming GA Petrie JR Bergenstal RM , et al. Diabetes digital app technology: benefits, challenges, and recommendations. A consensus report by the European Association for the Study of Diabetes (EASD) and the American Diabetes Association (ADA) Diabetes Technology Working Group. Diabetes Care 2020; 43: 250–260. 10.2337/dci19-0062 31806649

[bibr16-20552076231220791] Palta P Huang ES Kalyani RR , et al. Hemoglobin A1c and mortality in older adults with and without diabetes: results from the National Health and Nutrition ExaminationSurveys (1988–2011). Diabetes Care 2017; 40: 453–460. 10.2337/dci16-0042 28223299 PMC5864101

[bibr17-20552076231220791] Walle AD Ferede TA Shibabaw AA , et al. Willingness of diabetes mellitus patients to use mHealth applications and its associated factors for self-care management in a low-income country: an input for digital health implementation. BMJ Health Care Inform 2023; 30: e100761. doi: 10.1136/bmjhci-2023-100761 PMC10230908 37236653

[bibr18-20552076231220791] Mourão LF Marques ADB Moreira TMM , et al. Mobile applications to promote diabetic foot care: scoping review. Rev Eletr Enferm 2022; 24: 1–8.

[bibr19-20552076231220791] Diabetes Association of the Republic of China (Taiwan). Executive summary of the DAROC clinical practice guideline for diabetes care – 2018. J Formos Med Assoc. 2020; 119: 577–586. 10.1016/j.jfma.2019.02.016 30952480

[bibr20-20552076231220791] Dincer B Bahçecik N . The effect of a mobile application on the foot care of individuals with type 2 diabetes: a randomised controlled study. Health Educ J 2021; 80: 425–437. 10.1177/0017896920981617

[bibr21-20552076231220791] Laanpere M . Recommendations on assessment tools for monitoring digital literacy within UNESCO’s digital literacy global framework. United Nations Educational Scientific and Cultural Organisation. 2019; UIS/2019/LO/IP/56. https://unesdoc.unesco.org/ark:/48223/pf0000366740

[bibr22-20552076231220791] Lee SH Chen SM . The effects of the multimedia foot care program for patients with diabetes. 2021 Taiwan Association of Nurse Practitioners Conference, Posters Presentation. 2021; https://www.tnpa.org.tw/en/

[bibr23-20552076231220791] Chin YF Huang TT . Development and validation of a diabetes foot self-care behavior scale. J Nurs Res 2013; 21: 19–25. 10.1097/jnr.0b013e3182828e59 23407334

[bibr24-20552076231220791] Najafi B Mishra R . Harnessing digital health technologies to remotely manage diabetic foot syndrome: a narrative review. Medicina (Kaunas) 2021; 57: 377. doi: 10.3390/medicina57040377 33919683 PMC8069817

[bibr25-20552076231220791] Health literacy material design guidelines from Health Promotion Administration, Ministry of Health and Welfare Taiwan, 2017, https://www.hpa.gov.tw/File/Attach/7999/File_7544.pdf

[bibr26-20552076231220791] Sharoni SKA Rahman HA Minhat HS Shariff-Ghazali SS . The effects of self-efficacy enhancing program on foot self-care behaviour of older adults with diabetes: a randomised controlled trial in elderly care facility, Peninsular Malaysia. PlosOne 2018; 13: e0192417. 10.1371/journal.pone.0192417 PMC5849313 29534070

[bibr27-20552076231220791] Hirpha N Tatiparthi R Mulugeta T . Diabetic foot self-care practices among adult diabetic patients: a descriptive cross-sectional study. Diabetes Metab Syndr Obes: Targets Ther 2020; 13: 4779–4786. 10.2147/DMSO.S285929 PMC7723031 33304103

[bibr28-20552076231220791] Abredari H Bolourchifard F Rassouli M , et al. Health locus of control and self-care behaviors in diabetic foot patients. Med J Islam Repub Iran 2015; 29: 283. https://www.ncbi.nlm.nih.gov/pmc/articles/PMC4764266/26913246 PMC4764266

